# 

**Published:** 1879-01

**Authors:** 


					﻿Whole Number,
CCCCIV.
January, 1879.
Number 1,
Vol. XXXVIII.
,	THE
CHICAGO
MEDICAL
J ournal and Examiner
EDITORS:
WILLIAM H. BYFORD, A.M., M.D..
JAS. NEVINS HYDE, A.M., M.D. FERD. C. HOTZ, M.D.
E. FLETCHER INCALS, M.D.
CHICAGO:
PUBLISHED BY TIIE CHICAGO MEDICAL PRESS ASSOCIATION.
188 Clark Street.
3TRead the Notice of this Journal among Advertisements.
				

